# T2-weighted spoiled gradient echo MRI for forensic age estimation: a study on knee growth plates

**DOI:** 10.1007/s00414-024-03345-6

**Published:** 2024-10-12

**Authors:** Oguzhan Ekizoglu, Ali Er, Elif Hocaoglu, Mustafa Bozdag, Silke Grabherr

**Affiliations:** 1 Department of Forensic Medicine, Tepecik Training and Research Hospital, Güney Mahallesi 1140/1 Yenisehir, Konak, Izmir, Turkey; 2 University Centre of Legal Medicine Lausanne-Geneva, Geneva University Hospital and University of Geneva, Geneva, Switzerland; 3 Department of Radiology, Tepecik Training and Research Hospital, Izmir, Turkey; 4 Department of Radiology, Bakırkoy Dr.Sadi Konuk Training and Research Hospital, Istanbul, Turkey

**Keywords:** Forensic age estimation, MRI, MERGE sequence, Distal femoral epiphysis, Proximal tibial epiphysis, Growth plate closure, Skeletal maturity

## Abstract

The timing of growth plate fusion is a key indicator for age estimation and is primarily used in forensic investigations. On the other hand, non-ionizing techniques such as MRI are being developed to provide safer and more ethical evaluations in forensic casework. This study aims to evaluate the closure process of growth plates in the distal femoral and proximal tibial epiphyses using Multiple Echo Recombined Gradient Echo (MERGE) MRI sequences and provide age estimation data based on staging methods for forensic purposes. We retrospectively analyzed 559 patients (294 males, 265 females, aged 8–25 years) diagnosed with trauma and knee pain at Tepecik Training and Research Hospital from 2016 to 2019. MRI scans were performed using a 1.5-T system with MERGE sequences and evaluated by two observers using a new staging system. Observer agreement was assessed using Cohen’s κ test, yielding high agreement values (κ > 0.8). Positive correlations were found between age and ossification stages (*p* < 0.001). Minimum age thresholds for stages 5a and 5b of the distal femoral epiphysis were 16 and 18 years for females and 17 and 19 years for males, respectively. For the proximal tibial epiphysis, the minimum ages for stages 5a and 5b were 15 years for females and 17 years for males. The MERGE sequence provides a viable method for assessing skeletal maturity in living individuals with significant ethical advantages due to non-ionizing radiation. This study supports the potential application of the MERGE sequence in forensic age estimation, demonstrating high observer agreement and consistency. Future research should focus on comparing different sequences and populations to enhance the methodology’s applicability.

## Introduction

Accurate age estimation in legal and forensic contexts is crucial for protecting individuals’ rights and ensuring justice, as age is a fundamental criterion in determining both criminal responsibility and social rights. This is especially critical concerning the rights of migrants. Cross-border migration affecting Europe remains one of the most significant issues for the international community [1]. The European Union (EU) border security agency, Frontex, reported an 86% increase in irregular migration to the EU between January and July 2022 compared to the same period in the previous year [2]. According to Frontex, a total of 155,090 irregular migrants crossed EU borders in the first half of the year. With 34,570 migrants arriving in July alone, irregular migration traffic was 65% higher than in July of the previous year [2]. For European countries, determining the age of migrants is a starting point for the application of major social, legal, and civil rules. The importance of classifying migrants as children or adults is particularly significant for determining and protecting children’s rights [3]. Furthermore, age estimation is crucial for any criminal or legal case involving the migrant, depending on their status. On the other hand, attempts by migrants to manipulate their age to appear younger to obtain different social and legal rights are also observed [4].

The German Society of Legal Medicine’s (DGRM) Working Group on Forensic Age Diagnostics (AGFAD) and the European Association of Forensic Anthropology have provided recommendations for determining an individual’s age. These recommendations include clinical evaluation, a combination of physical and radiographic examinations of the left hand, a dental examination, and an orthopantomography evaluation. If ossification of the hand is complete, the degree of clavicular ossification should be evaluated by conventional radiography or computed tomography [5, 6]. It should be noted that the recommended methods for living individuals, especially during childhood, involve exposure to X-ray radiation [3]. Since forensic age estimation is not a medical indication, applying these methods to individuals requires a specific legal basis [7]. To mitigate the ethical challenges posed by this situation, increasing research has focused on non-ionizing radiation methods in recent years. When considering the advantages of cross-sectional analysis, MRI has emerged as a prominent tool in these analyses [8–12]. A smaller number of studies have also investigated the use of ultrasound for forensic age estimation [15–17].

Most studies on forensic age estimation using MRI focus on examining the knee joint. Other studies have examined the clavicle, wrist, shoulder, and ankle joints [8–15]. These studies primarily rely on Schmeling and Kellinghaus staging, as well as the Dedouit and Vieth staging methodologies [9, 18–20]. These methodologies utilize different MRI weights (T1, STIR, FSE PD, etc.) based on T1 and T2 sequences, and efforts are being made to develop methods using data provided by different MRI weights [8–20].

In recent years, a gradient-echo sequence has been developed, which combines multiple gradient echoes, using early echoes to increase the signal-to-noise ratio and later echoes to enhance image contrast [21]. This sequence is known as MERGE (Multiple Echo Recombined Gradient Echo) for GE Healthcare, MEDIC (Multiecho Data Image Combination) for Siemens Healthcare, and MFFE (Multiecho Fast Field Echo) for Philips Healthcare. MERGE, developed by GE Healthcare, is a T2* weighted sequence designed for spinal and musculoskeletal imaging [22]. Understanding the potential impact of different MRI sequences on forensic age estimation can contribute to increasing data based on MRI examination for age estimation. Therefore, this study aims to evaluate the process of growth plate closure in the distal femoral epiphysis and proximal tibial epiphysis using MERGE MRI images and provide age data based on the staging method for forensic age estimation.

## Materials and methods

This study was carried out at Tepecik Training and Research Hospital in Izmir, Turkey. We retrospectively evaluated the clinical and radiological data of patients diagnosed with trauma and knee pain admitted to different clinics of the hospital between 2016 and 2019. The study protocol was approved by the hospital’s ethical board. After reviewing the medical records and MRI scans, information on socioeconomic status and ethnicity was not included in the medical records; therefore, no assessment was made in this regard. Patients were excluded if they had any knee pathology (such as tumors, fractures, infections, surgical fixation, or bone marrow edema), neoplastic disease, radiation or chemotherapy. They were also excluded if the obtained images were of insufficent quality do to typical MRI artifacts. In this study, 61 patients with bone marrow edema (14), surgical fixation (7), hypothyroidism (2), and MRI with motion artifacts (38) were excluded. Data were obtained for 559 patients (294 males and 265 females) with an age range of 8–25 years.

All examinations were performed using the following MRI systems: a 1.5-T system (Optima MR360, GE Healthcare) at Tepecik Training and Research Hospital with coronal T2*-weighted spoiled gradient echo images (MERGE) (TR, 596 ms; TE, 5 ms; slice thickness, 3.5 mm; FOV, 20 cm; flip angle, 20; acquisition time, 3.39 min). A knee coil was used to evaluate the skeletal maturation of the distal femoral and proximal tibial epiphyses. Two observers interpreted the images at radiological monitors with resolutions of 2048 × 1536 pixels (RadiForce RX350, EIZO, Japan). Each MRI scan was evaluated twice by an experienced expert in forensic medicine (R1) and an experienced radiologist (R2). All images were re-evaluated after four weeks without prior knowledge of the staging results. In this study, we have introduced modifications to the existing staging system [20] by adding sub-stages 4a, 4b, 5a, and 5b. as shown in Table 1.

**Table 1:** Definition of grading system

**Fig. 1:**
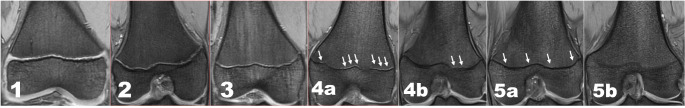
Multiple Echo Recombined Gradient Echo (MERGE) MRI sequences in coronal orientation on knee MRI: stage 1 to stage 5b for distal femoral epiphysis. The arrows highlight hyperintense areas for stages 4a and 4b, and hypointense areas for stage 5a

**Fig. 2:**
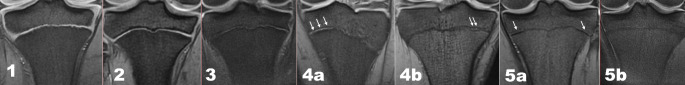
Multiple Echo Recombined Gradient Echo (MERGE) MRI sequences in coronal orientation on knee MRI: stage 1 to stage 5b for proximal tibial epiphysis. The arrows highlight hyperintense areas for stages 4a and 4b, and hypointense areas for stage 5a

**Stage 1** A continuous horizontal cartilage layer thicker than 1.5 mm was apparent between the junctions ofthe metaphysis and the epiphysis, and the cartilage was multilaminar in appearance (Figs. 1 and 2). The multilaminar appearance was observed as decreased signal intensity in the upper layer, increased signal intensity in the middle layer, and decreased signal intensity in the lower layer.
**Stage 2** A continuous horizontal linear cartilage signal intensity was present between the metaphysis and the epiphysis, with a thickness greater than 1.5 mm, with increased signal intensity but without a multilaminar appearance (Figs. 1 and 2).
**Stage 3** A continuous horizontal linear cartilage signal intensity was present between the metaphysis and the epiphysis, with a thickness less than 1.5 mm and increased signal intensity (Figs. 1 and 2).
**Stage 4** Discontinuous horizontal linear cartilage signal intensity was present between the metaphysis and the epiphysis, with a thickness less than 1.5 mm and discontinuously increased signal intensity (Figs. 1 and 2). ** Stage 4a** Discontinuous horizontal linear thin cartilage signal intensity was present over one third of the distance between the metaphysis and the epiphysis ** Stage 4b** Discontinuous horizontal cartilage signal intensity was present one third or less of the distance between the metaphysis and the epiphysis
**Stage 5** Signal intensity between the metaphysis and the epiphysis was not increased (Figs. 1 and 2). ** Stage 5a** Continuous or discontinuous hypointense line visible ** Stage 5b** Hypointense line is not visible

### Statistical analyses

Statistical analyses were performed using the Statistical Package for the Social Sciences (SPSS) software (ver. 17; IBM Corporation, Armonk, NY, USA). Values were expressed as means or medians, with standard deviations (SDs), lower and upper quartiles, and minimum and maximum values, where applicable. Correlations between age and ossification stage were evaluated by Spearman’s correlation analysis. The Mann-Whitney U test was used for comparisons between sexes. Statistical significance was defined as a p-value < 0.01. Cohen’s κ test was used to assess the level of agreement between observers and time points. The κ values, weighted κ values, and agreement rates were calculated. The κ values were interpreted using the system developed by Altman [23].

## Results

This study evaluated images from 559 patients (294 males and 265 females) with an age range of 8–25 years. The mean age of the male patients was 17.97 ± 4.12 years, and that of the female patients was 17.30 ± 3.81 years. Intra- and interobserver agreement for the assessment of the distal femoral and proximal tibial epiphysis were calculated separately. Intraobserver agreement for the distal femoral epiphysis was κ = 0.889, and interobserver reliability was κ = 0.867. The intraobserver agreement for the proximal tibial epiphysis was κ = 0.893, and the interobserver reliability was κ = 0.806. Thus, for both the distal femoral and proximal tibial epiphysis, the intra- and inter-observer assessments showed very good repeatability and consistency of the method. Spearman’s rank correlation analysis showed a significant positive correlation between age and ossification stage of both epiphyses (distal femoral: all subjects: rho = 0.793, *p* < 0.001; male: rho = 0.826, *p* < 0.001; female: rho = 0.753, *p* < 0.001; proximal tibial epiphysis: all subjects: rho = 0.714, *p* < 0.001; male: rho = 0.776, *p* < 0.001; female: rho = 0.645, *p* < 0.001). Statistical analysis of sex differences revealed significant differences for the distal femoral epiphysis at stages 2, 4a, 5a, and 5b (*p* < 0.01) but not for stages 1 (*p* = 0.104), 3 (*p* = 0.850), and 4b (*p* = 0.182). For proximal tibial epiphyses, a significant difference was found for stages 5a and 5b (*p* < 0.01) but not for other stages (p values > 0.01).

The stages of ossification for both epiphyses in our sample were estimated. The MRI findings for ossification stages 1 to 5b observed in both epiphyses are shown in Figs. 1 and 2. The age distribution for both sexes is presented in Table 2. Among males, ages ranged from 8 to 25 years, with the highest frequency observed at ages 17 and 18, with 38 and 41 subjects, respectively. For females, ages ranged from 8 to 25 years, with the highest frequency observed at ages 14 and 19, with 34 and 34 subjects, respectively.

**Table 2:** Age distribution of male and female subjects

Age (years)	Male (*N*)	Female (*N*)
8	2	2
9	1	3
10	7	9
11	15	6
12	13	16
13	13	14
14	27	34
15	14	16
16	26	16
17	38	29
18	41	22
19	16	34
20	8	20
21	12	23
22	6	14
23	8	9
24	5	11
25	13	16
Total	265	294

For the distal femoral epiphysis, the minimum ages at all stages for both sexes were analyzed. In females, the youngest ages observed were 8.08 years at stage 1, 11.42 years at stage 2, 13.51 years at stage 3, 14.12 years at stage 4a, 15.42 years at stage 4b, 16.00 years at stage 5a, and 18.00 years at stage 5b. In males, the youngest ages observed were 8.08 years at stage 1, 12.17 years at stage 2, 14.00 years at stage 3, 15.05 years at stage 4a, 16.42 years at stage 4b, 17.19 years at stage 5a, and 19.08 years at stage 5b.

For the proximal tibial epiphysis, the analysis revealed the minimum ages at each stage for both sexes. In females, the youngest ages were 8.08 years at stage 1, 11.00 years at stage 2, 13.00 years at stage 3, 13.30 years at stage 4a, 14.25 years at stage 4b, 15.00 years at stage 5a, and 15.00 years at stage 5b. In males, the youngest ages were 8.08 years at stage 1, 11.08 years at stage 2, 13.08 years at stage 3, 14.33 years at stage 4a, 15.10 years at stage 4b, 16.08 years at stage 5a, and 17.00 years at stage 5b. The minimum and maximum ages at which the stages were seen, together with the lower and upper quartiles, medians, means, and standard deviations of all the parameters, are shown in Tables 3 and 4.

**Table 3:** Minimum and maximum ages, with means ± SDs, lower quartiles (LQ), upper quartiles (UQ), medians, and sample sizes (N) at all stages of distal femoral epiphysis

Sex	Stage	*N*	Min	Max	Mean ± SD	LQ	UQ	Median
Female	1	33	8.08	14.85	11.78 ± 1.55	10.92	12.92	12.03
	2	15	11.42	13.83	12.28 ± 0.85	11.75	12.75	11.84
	3	20	13.51	15.75	14.61 ± 0.69	14.25	14.92	14.68
	4a	32	14.12	21.99	16.24 ± 2.11	14.65	17.53	15.18
	4b	17	15.42	23.20	17.66 ± 2.20	15.83	18.94	16.82
	5a	121	16.00	25.98	19.77 ± 3.06	17.30	21.91	18.44
	5b	27	18.00	24.42	18.83 ± 1.24	18.25	18.88	18.58
Male	1	50	8.08	16.47	12.37 ± 2.04	10.52	13.86	12.92
	2	20	12.17	14.92	13.55 ± 0.96	12.50	14.27	13.79
	3	24	14.00	16.83	14.70 ± 0.57	14.33	14.94	14.54
	4a	29	15.05	21.88	16.98 ± 1.53	15.83	17.42	16.75
	4b	17	16.42	25.18	18.56 ± 2.46	16.92	19.58	17.42
	5a	124	17.19	25.98	21.16 ± 2.65	18.81	23.30	21.14
	5b	30	19.08	24.50	20.36 ± 1.31	19.50	20.42	19.96

**Table 4:** Minimum and maximum ages, with means ± SDs, lower quartiles (LQ), upper quartiles (UQ), medians, and sample sizes (N) at all stages of proximal tibial epiphysis

Sex	Stage	*N*	Min	Max	Mean ± SD	LQ	UQ	Median
Female	1	26	8.08	14.85	11.75 ± 1.64	10.73	12.92	12.12
	2	19	11	13.83	12.06 ± 0.82	11.44	12.62	11.92
	3	8	13	14.92	14.02 ± 0.79	13.55	14.78	13.92
	4a	26	13.3	22.81	15.84 ± 2.64	13.81	17.05	14.81
	4b	15	14.25	23.2	16.07 ± 2.78	14.59	15.78	14.83
	5a	139	15	25.98	19.23 ± 3.20	16.67	21.59	18.45
	5b	32	15	24.42	17.46 ± 1.88	16.18	18.19	17.33
Male	1	44	8.08	16.47	12.44 ± 2.13	10.42	13.91	12.94
	2	23	11.08	14.58	12.86 ± 1.15	11.92	13.92	12.73
	3	19	13.08	15.57	13.81 ± 0.68	13.29	14	13.72
	4a	27	14.33	19.77	15.70 ± 1.29	14.79	16.73	15.33
	4b	6	15.1	17.17	16.38 ± 0.79	15.95	16.87	16.66
	5a	145	16.08	25.98	20.75 ± 2.82	18.47	22.47	20.73
	5b	30	17	24.5	19.24 ± 1.72	17.92	19.87	18.91

## Discussion

This study represents the first MRI study on the use of the MERGE sequence for evaluating the developmental stages of the knee epiphyses for age estimation in living individuals. While the developmental staging system developed by Dedouit et al. [20] was used for age estimation, two separate sub-stages were defined for stages 4 and 5. Minimum ages for stages 4a and 4b were determined to be 14 and 15 years for females and 15 and 16 years for males, respectively, while minimum ages for stages 5a and 5b were 16 and 18 years for females and 17 and 19 years for males, respectively. For the distal tibia, minimum age values for stages 5a and 5b were determined to be 15 years for females and 17 years for males.

Observer agreement data is crucial for observational studies, especially for methodologies being used for the first time. Since this study was conducted using a new MRI sequence to evaluate knee epiphyses, all data were assessed for intra- and interobserver agreement, both of which were found to be high. As highlighted by Wittschieber et al. [24], observer experience can affect the reliability of results. The high agreement values may have been influenced by the experience of the center where the study was conducted and the observers analyzing the images in using different MRI sequences and staging methodologies for forensic age estimation in the past. Therefore, for the repeatability of the technique, future studies should involve different observers and populations.

Ethical concerns regarding radiation exposure in forensic age estimation primarily highlight MRI-based methodologies. Different sequences of MRI are used to evaluate epiphyseal development in forensic age estimation. Data obtained from these evaluations are classified using different staging methodologies. Studies exist on the use of T1-SE, T1-TSE, and T1-VIBE sequences for evaluating ossification in different epiphyseal areas [25]. The staging methodologies developed from T1-based MRI analyses are typically performed using the Schmeling and Kellinghaus staging systems [18, 19]. Unlike T1-weighted evaluations, T2 and FSE PD-weighted MRI are used to evaluate the developmental stages of hyaline cartilage. Dedouit et al. [20] first defined a 5-stage methodology in their analyses of knee epiphyses. The use of the Dedouit staging system with FSE PD and T2-weighted MRI has been employed to examine the proximal tibia, distal femur, proximal humerus, and distal radius epiphyses [8, 26–29]. Each MRI weight produces images based on the designed sequence, and the data from these images are classified using different staging systems, resulting in varying lower age limits for each combined method for forensic age estimation. This is particularly important for effectively determining significant age limits in forensic age estimation [28]. Age estimation based on the MERGE MRI evaluation of the distal femoral and proximal tibial epiphyses has not been tested in humans until now. This suggests that testing this sequence on knee epiphyses could provide additional information to past knee MRI studies and new data on minimum age limits.

This retrospective study has several limitations. Similar to many retrospective age estimation studies in recent years, an uneven age distribution is the primary limitation. Minimum age thresholds may be influenced by this, but the study’s reliance on retrospective clinical data makes overcoming this limitation challenging. Nonetheless, the data obtained may be strengthened by different population assessments over the years. Prospective studies with balanced age distributions may be more suitable for forensic age estimation, and more studies are being conducted despite the specific challenges associated with prospective methodologies [9, 11].

Given the frequent study of MRI evaluation of knee epiphyses for age estimation, considering past studies’ results can be beneficial. This study, in particular, could take into account the data from other T2-weighted sequences. Forensic age studies based on population screenings have identified different minimum age limits using various methodologies. The data obtained in this study can be compared to those from Kvist et al. [30], who used the MERGE sequence and a defined staging system for evaluating knee epiphyses. Kvist et al. used a staging system with minor modifications of Dedouit, Schmeling, and Kellinghaus staging systems. Although the staging system is not identical to this study, the stages are similar in defining the developmental stages of hyaline cartilage. Minimum ages for stage IV (The cartilage is not continuous) for the distal femur were 14 years for both males and females, and for stage V (fusion completed), the minimum ages were 14 and 16 years for females and males, respectively. For the proximal tibia, minimum ages for stages IV and V were 14 years for females and 14 and 15 years for males, respectively. Kvist’s study had a minimum age of 14 years for the population assessed, which may limit the data for stage IV. Additionally, Kvist et al. used MRI from three different devices. While the sequences for cartilage evaluation are similar, the sequence settings differ and can result in different image qualities. This creates a significant limitation regarding the data obtained from the cartilage sequences for the entire population being assessed for forensic age estimation. Other studies on forensic age estimation using hyaline cartilage evaluation include Dedouit et al. [20], who reported minimum age limits of 16 and 17 years for stage IV and 22 years for stage V in both sexes using FSE PD-weighted MRI. In a study using T2-weighted MRI by Ekizoglu et al. [29], the minimum age limit for stage V was 21 years for females, with other age limits similar to those in the Dedouit study. On the other hand, Uygun et al. [27] reported minimum age limits of 14 and 15 years for stages 4 and 5 for females and 15 and 16 years for males in a study conducted with the Turkish population using FSE PD. The study population represented a region in southern Turkey, known for its hot climate and demographic diversity with numerous ethnic backgrounds. This diversity may have influenced the lower minimum age limits. However, the study lacked data on ethnic and socioeconomic distribution, making it impossible to draw definitive conclusions.

Vieth et al. [9] conducted a forensic age estimation study using T1-weighted (T1-w) turbo spin-echo (TSE) and T2-weighted (T2-w) TSE sequences with fat suppression by spectral pre-saturation with inversion recovery (SPIR). This study used a 5-stage (Stages 2–6) analysis of the distal femoral epiphysis, with minimum age limits of 14.8 and 15.7 years for females and males for stage 5, respectively, and 20.6 and 21.2 years for stage 6, respectively.

The differences between past studies and this study can be attributed to the MRI technique applied, staging methodology, and population-specific characteristics. While ethnicity is not considered a factor influencing ossification, a low socioeconomic level is one of the factors affecting delayed ossification [31, 32]. This retrospective study lacked data on socioeconomic or ethnic backgrounds, making it impossible to determine the influence of socioeconomic status on the study results. Regarding the differences in MRI techniques, one factor that could affect image evaluation is the strength of the magnetic field. Theoretical advantages of 3 Tesla MRI applications include higher resolution images, higher contrast, and shorter scan times. Although forensic age estimation studies using 1.5T and 3T magnetic fields exist, no study has evaluated the effect of magnetic field differences on age estimation, especially concerning T2-weighted sequences. Only Saint-Martin et al. [33] argued that the magnetic field strength has no impact on forensic age estimation in their study on distal femoral epiphyses using T1-weighted MRI. However, the higher magnetic field strength of 3 Tesla MRI scanners might lead to better image quality compared to 1.5 Tesla scanners, potentially offering a higher signal-to-noise ratio and improved spatial resolution.

Other factors specific to MRI evaluations include artifacts. Parameters of the scan, specific tissue characteristics, data acquisition, and data reconstruction are major factors influencing artifact formation [34–36]. Numerous technical adjustments are made by prioritizing clinical imaging requirements [37]. The MRI images evaluated in this study were based on clinical imaging and parameters adjusted to prioritize pathological imaging. The MERGE sequence can offer high-resolution, isotropic T2*-weighted images of hyaline cartilage with good SNR and fat saturation properties, making it well-suited for assessing the developmental characteristics of hyaline cartilage as defined by Dedouit et al. However, accurate evaluation requires the clear visualization of hyperintense and hypointense cartilage lines and determining whether cartilage thickness is 1.5 mm, which could be challenging for inexperienced observers. Past forensic age estimation studies using FSE PD and T2-weighted MRI have reported low observer error rates. However, since these studies primarily involved observers experienced in forensic age estimation and MRI use, the potential challenges for new studies cannot be excluded. The MERGE sequence helps highlight specific tissue characteristics such as fat-water separation and hyaline cartilage. With high contrast and resolution, it provides clearer boundaries and brighter images for assessing hyperintense areas, thus being more advantageous for evaluating the continuity of the hyperintense area and measuring 1.5 mm thickness. These advantages in evaluation could contribute to identifying lower minimum age limits than those seen in FSE PD studies, allowing earlier and clearer visualization of hyperintense areas not clearly imaged with FSE PD. However, assessing the extent to which hyperintense areas are influenced by potential artifacts may be impossible. Although no artifacts were reported after excluding cases with motion artifacts in this study, the retrospective nature of our study made it impossible to test the potential effects of sequence design, coils, and gradients. Additional studies with various sequences, scanning procedures, and equipment are required to reduce artifact impacts and improve image quality [34–36]. Chemical shift artifacts, which can affect hyperintense area evaluations in the epiphyseal region, can be reduced by changing the direction of frequency and phase encoding gradients, using fat suppression, or increasing bandwidth [34]. Future prospective studies could identify potential causes and correction methods for artifacts.

## Conclusion

The use of the MERGE sequence for MRI evaluation of knee epiphyses can provide supporting data for forensic age estimation, with minimum age limits of 17 and 19 years for males and 16 and 18 years for females. This study demonstrates that using the MERGE sequence in MRI provides a viable method for assessing the skeletal maturity of both the distal femoral and proximal tibial epiphyses in living individuals. This non-ionizing imaging method offers a significant ethical advantage and an alternative methodology to meet the growing demand for less invasive diagnostic tools in forensic applications. High intra- and interobserver agreement highlights the method’s consistency and reliability, while the effects of varying experience levels require further investigation. Future studies can compare or combine methodologies with different sequences and assess the applicability of MERGE MRI to other skeletal regions to support its potential use in forensic applications.

## Data Availability

The datasets used and/or analyzed during the current study are available from the corresponding author on reasonable request.
